# Advances and Challenges in Bacterial Spot Resistance Breeding in Tomato (*Solanum lycopersicum* L.)

**DOI:** 10.3390/ijms21051734

**Published:** 2020-03-03

**Authors:** Pragya Adhikari, Tika B. Adhikari, Frank J. Louws, Dilip R. Panthee

**Affiliations:** 1 Department of Horticultural Science, North Carolina State University, Raleigh, NC 27695, USA; adpragya@illinois.edu (P.A.); fjlouws@ncsu.edu (F.J.L.); 2 Department of Entomology and Plant Pathology, North Carolina State University, Raleigh, NC 27695, USA; tbadhika@ncsu.edu

**Keywords:** bacterial spot, genome editing, host resistance, resistance breeding, transgenics, *Xanthomonas*

## Abstract

Bacterial spot is a serious disease of tomato caused by at least four species of *Xanthomonas*. These include *X. euvesicatoria* (race T1), *X. vesicatoria* (race T2), *X. perforans* (races T3 and T4), and *X. gardneri,* with the distinct geographical distribution of each group. Currently, *X. gardneri* and *X. perforans* are two major bacterial pathogens of tomato in North America, with *X. perforans* (race T4) dominating in east-coast while *X. gardneri* dominating in the Midwest. The disease causes up to 66% yield loss. Management of this disease is challenging due to the lack of useful chemical control measures and commercial resistant cultivars. Although major genes for resistance (*R*) and quantitative resistance have been identified, breeding tomato for resistance to bacterial spot has been impeded by multiple factors including the emergence of new races of the pathogen that overcome the resistance, multigenic control of the resistance, linkage drag, non-additive components of the resistance and a low correlation between seedling assays and field resistance. Transgenic tomato with *Bs2* and *EFR* genes was effective against multiple races of *Xanthomonas*. However, it has not been commercialized because of public concerns and complex regulatory processes. The genomics-assisted breeding, effectors-based genomics breeding, and genome editing technology could be novel approaches to achieve durable resistance to bacterial spot in tomato. The main goal of this paper is to understand the current status of bacterial spot of tomato including its distribution and pathogen diversity, challenges in disease management, disease resistance sources, resistance genetics and breeding, and future prospectives with novel breeding approaches.

## 1. Introduction

The cultivated tomato (*Solanum lycopersicum* L.) is an important vegetable crop in the United States and worldwide. Tomato is also a model crop to study fleshy fruit development and plant-pathogen interactions. Every year, tomato is inflicted with several bacterial, fungal, and viral diseases, including bacterial spot limiting its production. Bacterial spot (BS) disease is one of the major threats to tomato production that affects both fresh-market and processing tomatoes. The disease also occurs in pepper and other crops of the Solanaceae family [1]. Depending on the stage of infection and favorable climatic conditions, the disease can cause yield losses up to 66% [2,3]. In Florida, the tomato yield loss due to bacterial spot was estimated to be $3000 per acre ($7413 per ha) [4]. Likewise, ~$4 million to $12.5 million tomato yield losses have been reported in the Midwest of the USA [5]. Unfortunately, there are no commercial resistant tomato cultivars to this disease. The disease management relies on the spray of copper and antibiotics, although resistant bacteria to such chemicals have been reported from several regions. Host resistance is critical to manage the BS disease in tomato. Here, we review the diverse nature and species complex of the pathogen, available host resistance sources to BS disease, the genetic and breeding efforts deployed so far, including transgenic approach, and the challenges involved in BS disease resistance breeding. We also discuss the novel breeding strategies such as genomics-assisted breeding, effector-based breeding, genome editing, and high throughput phenotyping-based breeding that can be implemented to improve BS disease resistance in tomato. Our review will provide valuable information to tomato breeders and other researchers working in this area to improve tomato for BS disease resistance. 

## 2. Causal Pathogen, Race Structure, and the Distribution 

Bacterial spot of tomato is caused by at least four species of *Xanthomonas*: *X. euvesicatoria*, *X. vesicatoria*, *X. perforans*, and *X. gardneri* and four pathogenic races (T1, T2, T3, and, T4) are identified so far [6]. The T1 race has been reported in *X. euvesicatoria,* T2 race in *X. vesicatoria* and in *X. gardneri*, while T3 and T4 races are reported in *X. perforans* [6]. Mutation in effector genes *avrXv3* and *avrXv4* of races T3 and T4 belonging to *X. perforans* have also been identified and speculated to be race T5 [7]. However, isolation of race T5 under field conditions has not been reported yet. These pathogenic races are discovered based on hypersensitive responses (HR) on tomato differentials that include HI 7998 (T1), HI 7981 (T3), and LA716 (T4). However, no differential tomato germplasm is available showing HR response to race T2. This is why race T2 has been reported in both *X. vesicatoria* and *X. gardneri*.

*Xanthomonas euvesicatoria* was the only species, present as race T1, in Florida until 1991 before the *X. perforans* race T3 strain was reported. *Xanthomonas perforans* race T4 strain evolved in 1998 [7,8], and thereafter has been detected at higher numbers in field surveys in Florida. In a recent survey, only *X. perforans* race T4 were detected in the samples collected from different sites of Florida, and 94% of the sampled strains were *X. perforans* race T4 in North Carolina [3,9]. *Xanthomonas perforans* race T4 strains have also been reported in Louisiana [10]. *Xanthomonas gardneri* was found on a contaminated seed lot in 1991 and has since become widespread in the Midwest USA and Ontario Canada [11]. Both *X. gardneri* and *X. perforans* are predominant in Ontario, Canada [12]. *Xanthomonas gardneri* has been reported from tomato fields in Pennsylvania [13], and was responsible for the epidemics in the Midwest [5].

## 3. Plant-Pathogen Interactions and BS Disease Resistance

Host resistant *R* proteins mediate a response to effectors and activate plant defense responses such as effector-triggered immunity (ETI), which is characterized by localized cell death, termed as a hypersensitive response (HR) [14]. At least, 45 effectors have been reported in the bacterial spot. Among them *AvrBs2, XopD, XopF1, XopK, XopL, XopN, XopQ, XopR, XopX, XopZ1,* and *XopAD* represent the core effectors required during pathogenesis [1]. Tomato contains 355 *R* genes encoding the nucleotide-binding site-leucine-rich repeat (NB-LRR) proteins, and the role of each NB-LRR in the disease resistance is being studied [15]. Although only a few *R* genes against bacterial spot have been identified in tomato, these genes have not been fully utilized in the breeding program either because of a lack of markers or the evolution of new pathogen races.

Both the qualitative resistance and quantitative resistance have been identified in several tomato species in response to bacterial spot disease. The qualitative resistance characterized by HR has been identified to race T1 in *S. lycopersicum* accessions HI 7998 [16]; to race T3 in *S. lycopersicum* accessions HI 7981 and *S. pimpenellifolium* accessions PI128216 and PI 126932 [17,18]; to race T4 in *S. pennellii* accession LA716 [8]; and to *X. gardneri* in *S. pimpenellifolium* accessions LA2533, LA1936, and PI 128216 [19] (Table 1). Similarly, quantitative resistance against bacterial spot has been identified in *S. lycopersicum var. cerasiformae* accession PI 114490 (all races) [20,21]; *S. pimpenellifolium* accessions PI 126428, PI 340905-S, and PI 155372 to race T3 [17]; and *S. pimpenellifolium* accessions PI 128216 and PI 126932 to race T4 [17,18,20]. The availability of sources of resistance offers an opportunity to develop tomato cultivars resistant to existing races of these pathogens.

## 4. Genetics and Breeding Efforts to Improve the BS Disease Resistance So Far

Breeding tomato for resistance to BS disease has been challenging due to the evolution of new races of the pathogen limiting the effectiveness of known resistance genes, multigenic control of the resistance, and non-additive gene effects [38]. The genetics and breeding efforts to improve the BS disease resistance in tomato are summarized in Table 1. The HR-mediated-resistance in HI 7998 to race T1 is controlled by *Rx3* locus on chromosome 5 in multiple studies and is induced by the *avrRxv* effector of race T1 [22,23,24,25]. Initially, the marker Rx3-L1 was reported to be associated with the *Rx3* loci [25,40]. However, using a complex breeding population, Sim, et al., [26] identified SP5 associated with *Rx3* loci. The qualitative resistance to race T2 has not been reported so far. The HR-mediated-resistance in HI 7981, PI 126932, and PI 128216 to race T3 is controlled by a common locus *Rx4/Xv3* [33,41] that acted on a bacterial effector *avrXv3* [7,8]. The *Rx4* locus was fine mapped exactly at the pcc12 marker site and was of NBS-LRR class of resistance gene [32]. The resistance locus differed from the susceptible locus by 6-bp insertion/deletion (InDel) and eight SNPs. The HR-mediated-resistance in LA716 to race T4 is controlled by *RxopJ4* (*Xv4*) resistant locus [8,36,37]. The resistance locus *RXopJ4* was mapped to a 190 kb segment on the long arm of chromosome 6 between the markers J350 and J352 [37]. However, *RXopJ4* locus in LA 716 is linked with several negative traits, including autogenous leaf necrosis, low fruit yield, and small fruit size, and *RXopJ4* heterozygotes showed slow and weak HR along with inconsistent phenotypes, limiting the utilization of LA716 resistance in the breeding program [37]. 

*Solanum lycopersicum var. cerasiformae* PI 114490 conferred quantitative resistance to race T2, T3, and T4 [20]. The resistance to T2 race is controlled by two genes on chromosome 11 with additive gene effects and is different from those genes controlling resistance to race T3 [27]. In addition, three advanced breeding lines Fla. 8326 (large-fruited), Fla. 8233 (large-fruited), and Fla., 8517 (plum) with race T3 and race T4 resistance have also been developed [35]. These three lines have HI 7998 (in all three), PI128216 (Fla. 8233 and Fla. 8517), PI 114490 (Fla. 8517), and PI 126932 (Fla.8326) in their pedigree [35]. Hutton, et al. [38] identified a major QTL on chromosome 11 associated with the T4 resistance in PI 114490 derived inbred backcross (IBC) populations and confirmed this QTL in the populations derived from the three breeding lines Fla. 8517, Fla. 8233, and Fla. 8326. However, none of the PI114490 derived lines recovered full resistance of PI114490. The donor source for QTL on chromosome 11 was HI 7998 in all three breeding lines, suggesting epistatic gene interaction in the HI 7998 derived lines that is absent in HI7998 [38]. 

Bhattarai, et al. [42] reported the moderate level of resistance against race T4 in *S. pimpinellifolium* derived breeding lines, including 74L-1W (2008), NC2CELBR, 081–12–1X-gsms, NC22L-1 (2008), and 52LB-1, in the field and greenhouse studies. Further work is needed to characterize the resistance observed in these lines and to evaluate if the observed resistance in these lines is sufficient to manage the disease before integrating the resistance in the cultivar. Three *S. pimpinellifolium* accessions PI 128216, LA2533, and LA1936 showed both HR-mediated-resistance in the greenhouse and field resistance to *X. gardneri* and *X. perforans* race T3 [19]. HR to *X. perforans* was observed before the HR to *X. gardneri.* Another study identified quantitative resistance to *X. gardneri* in two varieties- IZK Alya (cherry type) and Nikolina F_1_ (determinate large fruit type) [43]. Two genes, *Bs7* and *Bs3*, have been reported to confer resistance to *X. gardneri* avirulence genes *avrBs7* and *AvrHah1*, respectively in pepper, another member of the Solanaceae family [44,45].

## 5. Transgenic Resistance to Tomato BS Disease

Transgenic resistance is considered a promising tool because it eliminates linkage drag observed in the tomato cultivars developed from backcrossing. The pepper gene *Bs2* targeting the core effector *avrBs2* of *Xanthomonas* strains has been deployed in tomato through the transgenic approach. The *Bs2* transgenic tomato conferred resistance to all field strains of *Xanthomonas* in FL and increased the yield compared to non-transformed lines without any adverse effects in the transgenic tomatoes [46]. *Bs2* gene is safe for human consumption because *Bs2* protein belongs to one of the largest families of naturally occurring plant proteins (NB-LRR family), which has no known adverse effects on human health and has been widely consumed [3]. Therefore, *Bs2* transgenic tomatoes should be safe as long as they are selectable marker-free.

However, a mutation in *avrBs2* effector has been observed in rare *Xanthomonas* strains that could overcome the resistance conferred by the *Bs2* gene in the future [47]. This suggests that multiple targets for such core effectors are necessary to achieve durable resistance. Two recessive genes in pepper *bs5* and *bs6* conferred resistance to all races of BS in peppers [48]. Such recessive genes are predicted more durable than the dominant resistance genes as these genes do not involve in specific gene-for-gene interactions [48]. Identifying and cloning tomato homologs of *bs5* and *bs6* genes will enhance the tomato breeding efforts for BS disease resistance and provide targets for future transgenic or gene editing approaches.

Transgenic approaches could also allow integration of *Bs3* and *Bs7* genes from pepper into a tomato to achieve resistance against *X. gardneri*. Moreover, transgenic expression of elongation factor-Tu (EF-Tu) receptor (*EFR*), a pattern recognition receptor (PRR) from *Arabidopsis thaliana* also conferred broad-spectrum bacterial resistance, including *X. perforans* in tomato. However, *EFR* resistance was less effective compared to that conferred by the combination of *Bs2* and *EFR* genes or *Bs2* gene alone [49]. Therefore, deployment of *Bs2* gene in combination with other novel resistance genes would provide durable resistance to BS disease in tomato. Although the effectiveness of *Bs2* transgenic tomatoes to manage BS disease is successfully demonstrated in multiple field trials, the *Bs2* transgenic tomatoes could not be commercialized because of public concerns towards genetically engineered food products.

## 6. Novel Breeding Strategies to Enhance Tomato BS Disease Resistance 

Despite several breeding efforts, no commercial tomato cultivars resistant to BS are available yet. This emphasizes the necessity of novel strategies in breeding and biotechnology to achieve durable disease resistance in tomato for BS. In recent years, genomics and effectoromics have opened the pathway to identify *R* genes targeting the core effectors of the pathogens. For instance, effector genomics was employed to identify late blight *R* genes and pathogen recognition receptors in potato [50,51]. Similarly, comparative genomics has enabled the capacity to compare the genomes of closely related species and study the conserved genes among different genera within the same family. For example, comparative genomics allowed to isolate *R3a* gene in potato against the late blight pathogen [52]. Additionally, genome editing technology has enabled precise modification of the plant genome to improve disease resistance in crop plants such as in wheat to enhance powdery mildew disease [53], in citrus to improve citrus canker resistance [54], and in rice to improve bacterial blight [55]. High-throughput phenotyping (HTP) is another strategy to accelerate the resistance breeding, which enables accurate scoring and continuous monitoring of crop diseases. For instance, the quantitative virulence of *Z. tritici* that causes Septoria tritici blotch on wheat was assessed using the HTP-based automated image analysis [56]. In this study, HTP not only allowed to analyze the leaf disease symptoms but also quantify the pycnidia size and density. This kind of disease assessment is beyond the visual assessment alone. In summary, with all these new technologies available, it is expected that tomato breeding programs in the future will succeed in developing BS disease-resistant tomato cultivars.

### 6.1. Exploiting Tomato Genomic Resources

Tomato whole genome sequence was made available in 2012, which has been a great resource for the study of several traits, including bacterial diseases in tomato [57]. Recently, a pangenome of tomato has been made available and has added 4,873 genes not present in the reference genome [58]. Currently, sequences of several tomato genotypes are publicly available, which is useful for exploring potentially useful genes and the development of molecular markers (www.solgenomics.net). With the reduction of sequencing costs, there are reports of sequencing individual lines as per their necessity for the development of SNP markers or the identification of genes of interest. Sequencing of tomato genotypes and landraces and exploiting sequence polymorphisms in different tomato genotypes and landraces will shed new light on the genetics and molecular basis of BS resistance in tomato. For example, molecular markers (SNP and InDels) were developed based on whole-genome re-sequencing of two tomato lines (Caimanta and LA0722) and using those markers in a segregating population; eventually, BS disease resistance lines were developed [59]. Menda et al. [60] have reviewed the various genomics resources that can be used in tomato improvement.

### 6.2. Genomics-assisted Breeding Approaches (GBA)

Genomic-assisted breeding approaches (GBA) include marker-assisted selection (MAS), marker-assisted recurrent selection (MARS), marker-assisted backcrossing (MABC), and genomic selection (GS). Most of the genetic studies (QTL mapping) underlying BS disease resistance in tomato were based on the controlled crosses with limited recombinant events. Although association mapping has a higher mapping resolution to dissect the genetic basis of several quantitative traits, including disease resistance, the inbred nature of the species, low level of molecular diversity, and high genomic linkage disequilibrium (LD) have limited association mapping studies in tomato [61]. In recent years, GWAS has been adopted in tomato to dissect genetic variation of agronomical traits and fruit quality in part due to the availability of whole-genome sequence data, low sequencing cost allowing resequencing analysis, and availability of high-density SNP markers covering the whole genome [62,63]. The GWAS studies, in combination with the next-generation sequencing technologies, provide higher mapping resolution to detect the genetic region underlying the BS disease resistance and sequence variation underlying the region associated with the resistance. The markers associated with the disease-resistance can then be used in a breeding program via MAS and MABC. For instance, the use of InDel markers to select for the QTL region and a panel of SNP markers assayed on the KASP platform to select background genome selection allowed for the rapid development of NILs that are 95%–99% genetically identical, except for the QTL on chromosome 11 [64,65,66]. Also, cost-effective genotyping methods and high-density SNP markers facilitate genomic predictions of breeding values and selection via GS approach. Duangjit et al. [67] studied the potential of GS in tomato breeding programs to improve fruit quality traits in tomato. In the case of BS disease resistance, Liabeuf et al., [68] observed higher prediction accuracy through the GS model for resistance to *X. euvesicatoria* compared to phenotypic selection.

### 6.3. Effectors-based Breeding Strategy 

Effectoromics is a powerful tool to exploit core effectors from diverse groups of *Xanthomonas* and their matching target *R* genes in tomato [69]. Core effectors are crucial for pathogenicity and virulence. Therefore, the pathogens have a fitness cost to evolve the core effectors [70]. The detection of such core effectors and deployment of the resistance genes targeting those core effectors will improve the disease resistance and increase the resistance durability. The availability of pangenome assists in identifying multiple NLRs to recognize core effectors of *Xanthomonas,* causing BS in tomato. Advancement in the computational tools will facilitate the identification of candidate effectors, while resistance gene enrichment sequencing (RenSeq) will identify multiple NLRs that recognizes core pathogen effectors to achieve the disease resistance [53]. Both core effectors and NLRs can be transiently expressed in the tomato plant, and the HR phenotype indicates the recognition of effector genes by NLR. High throughput functional screening will further accelerate the identification of promising NLRs to recognize the core effectors and inform *R* gene deployment before the pathogen evolution [53].

Tomato breeding programs should, therefore, aim to identify and utilize tomato NLRs, recognizing the core effectors of *Xanthomonas spp*. Although pepper gene *Bs2* targeting *AvrBs2*, a core effector conserved among *Xanthomonas* species and races infecting tomato, has been deployed in tomato, it could not be utilized widely due to public concerns of transgenic tomatoes [46]. However, the other effectors such as *avrBsT*, *T3SS* belonging to *XopJ* effector family with a role in pathogen fitness under field conditions were detected among *X. perforans* and some strains of *X. euvesicatoria* and *X. vesicatoria* [71]. Therefore, *avrBsT* might be a suitable candidate to target *X. perforans* in the regions where this species is predominant. The breeding approaches targeting evolutionary conserved bacterial effector in combination with resistant gene pyramiding could provide durable resistance against BS disease.

### 6.4. Genome Editing 

Genome editing (GE) is a great tool that accelerates the trait improvement in a precise way in a breeding program if the genome sequences of the particular crop are available. The GE allows precise single gene introgression, gene knock out or gene modification to obtain the desired trait in the crops. GE offers two advantages over traditional breeding and transgenic methods in BS resistance breeding in tomato, which are i) GE overcomes the problem of linkage drag associated with the resistance genes such as in *RxopJ4* gene as observed in the traditional breeding method, and ii) GE avoids the random gene insertion and any foreign gene insertion in the crop genome unlike in transgenic approach. Unlike GM technology, GE techniques have not been regulated by the United States Department of Agriculture (USDA) and thus avoids regulatory issues. The plant community has three genome editing tools-zinc-finger nucleases (ZFNs), transcription activator-like effectors (TALE) nuclease system (TALEN), and clustered regularly interspaced short palindromic repeats/cas9 (CRISPR/Cas9). The GE has been utilized to improve disease resistance in several crops to many diseases, such as blast and bacterial blight in rice [55,72,73,74]. The potential of GE to insert multiple genes into a single locus was demonstrated by stacking two herbicide tolerance genes in maize [75]. Therefore, such technology can also be implemented in BS disease resistance breeding to integrate multiple BS resistance genes such as *Bs2*, *EFR*, *Bs3*, *bs5*, *bs6*, and *Bs7* from the pepper. This will allow simple inheritance of stacked genes in the breeding program.

More recently, CRISPR/Cas9 has gained more popularity than other GE systems because of simplicity, efficiency, and low off-target sites [76]. The potential of CRISPR/Cas9 to generate stable, highly specific, and heritable mutations in tomato plants without any off-targets has been demonstrated [77]. Also, CRISPR/Cas9 has been adopted in tomato to improve several traits including disease resistance such as powdery mildew [78] and bacterial speck [79]. Therefore, CRISPR/Cas9 could provide an alternate option to classical transgenic methods to develop BS disease resistance in future tomato breeding. The CRISPR/Cas9 technique would be ideal for integrating NLRs from wild species into cultivated tomato [53]. Another application of the CRISPR/Cas9 system in BS resistance breeding could be editing the *Bs2* and *EFR* syntenic genes in tomato to make it functional as the *Bs2* gene and *EFR* gene, so that tomato lines with these genes could be commercialized, unlike the transgenic *Bs2* tomato lines. In a study, CRISPR/Cas9 was used to inactivate the *Sl-DMR6–1* gene in tomato, which conferred resistance to multiple important pathogens of tomato, including *X. perforans* and *X. gardneri* [80]. Therefore, integrating *Bs2*, *EFR*, and *Sl-DMR6–1* mutation in tomato cultivars could provide a durable resistance to BS disease. To conclude, the potential of GE in improving BS disease resistance in tomato cannot be neglected. 

### 6.5. Phenomics-Assisted Breeding

Although much progress has been achieved in the genomics area, phenotyping is still lagging behind. Precise phenotyping is critical in the case of breeding for complex traits such as disease resistance, and the phenotypic data are the primary data in case of association studies. The inaccurate phenotyping might give false-positive results during QTL analysis and genomics-assisted breeding. The phenotyping of BS disease is usually done visually using a traditional numeric scale, which is subjective and might not be precise and accurate. 

In recent years, several high throughput phenotyping tools and phenomics platforms have been developed, which allow the collection of accurate and correct phenotypic data for genomics-assisted breeding and the identification of gene function [81]. The resources or projects for the phenomics studies in plants include International Plant Phenotyping Network (IPPN), European Plant Phenotyping Network (EPPN), German Plant phenotyping Network (DPPN), The Jülich Plant Phenotyping Centre (JPPC), PHENOME, the Australian Plant Phenomics Facility (APPF), Laboratory of Plant Ecophysiological Response to Environmental Stresses (LEPSE), Green Crop Network (GCN), mutant genotype and phenotype dataset, PhenoPhyte, and SciNetS [82,83]. 

For the quantification of diseases, images-based methods that rely on the reflectance of waves have been developed, such as analyzing the images in the visible spectrum, hyperspectral images, thermographic images, and chlorophyll fluorescence images [84]. Likewise, several automated and versatile disease phenomics platforms based on imaging techniques have been developed. For instance, the multi-sensor platforms Scanalyser^3DHT^ and Scanalyser FIELD are developed by the company LemnaTec (http://www.lemnatec.com) for the high-throughput phenotyping in the greenhouse and field, respectively. The American company Qubit Systems (http://qubitsystems.com/portal/) developed PlantScreen™ Conveyor Systems and PlantScreen™ Field Systems for greenhouse and field studies, respectively [84]. The image-based methods have been implemented to examine the diseases caused by the genus *Xanthomonas* in citrus [85] and bean [86].

Therefore, plant disease phenomics will not only allow us to collect BS disease data accurately or quantify the variation precisely, but also generate the reproducible data. This allows the accurate dissection of the underlying genetic architecture of BS disease resistance during QTL mapping and association mapping studies. This provides a better understanding of the mechanism of BS disease resistance in tomato and guides tomato breeders accordingly to improve BS disease resistance in tomato.

## 7. Conclusions

One of the current challenges in the BS disease resistance breeding program is to achieve durable resistance. Despite the continuous efforts to identify and introgression of resistance, it has been challenging to develop a BS disease-resistant cultivar for a long time because of the emergence of new races and species of the pathogen. The negative correlation between fruit quality and disease resistance has made the introgression of resistance even more challenging. In recent years, we have ample opportunities, as explained in the above sections, and can go beyond the classical breeding approaches to obtain broad-spectrum BS disease resistance against multiple species and races of the pathogen. The goal of improving disease resistance to BS disease in tomato can be achieved through the integration of available tomato genome resources, genomics tools, gene or genome editing tool, effectoromics, and plant disease phenomics tools. Employing multidisciplinary approaches seeks not only to improve disease resistance, but also to increase yield and improve fruit quality in tomato. 

## Figures and Tables

**Table 1:** Summary of breeding efforts for resistance to different races of *Xanthomonas,* causing bacterial spot disease in tomato.

Race Type	Resistance Genotype	Species ^a^	Resistant Genes ^b^	Chromosome ^c^	Bacterial Effector ^d^	Markers ^e^	References
T1 (before 1989)	HI 7998 (HR)	*S. lycopersicum*	*Rx3*	5	*avrRxv*	Rx3 -L1, SP5, TOM196 (SSR), TOM144 (SSR), COSOH57(SNP)	[16,18,22,23,24,25,26]
			*Rx2*	1			[16,22,23,24]
			*Rx1*	1			[16,22,23,24]
	PI114490	*S. lycopersicum var. cerasiformae*	quantitative	2,3,10,11			[21]
T2 (1989)	PI114490	*S. lycopersicum var. cerasiformae*	quantitative	2,3,10,11	deletion of 680 bp region of *avrRxv*		[20,21,22,27,28]
T3 (1992)	HI 7981 (HR)	*S. lycopersicum*	*Xv3*	11	*avrXv3*	cLEC-24-C3 (SNP), SL10029 (SNP)	[17,18,29,30]
	PI 128216 (HR)	*S. pimpenellifolium*	*Rx4*	11	*avrXv3*	pcc12	[17,31,32]
	PI 126932 (HR)	*S. pimpenellifolium*	*Rx4*	11	*avrXv3*		[18,33]
	LA716 (HR)	*S. pennelii*	*Xv4*		*avrXv4*		[8]
	LA 1589 (HR)	*S. pimpenellifolium*	*Rx_LA1589_*	11	*avrXv3*		[34]
	PI114490	*S. lycopersicum var. cerasiformae*	quantitative	2,3,10	*-*	-	[21]
	PI 126428		quantitative	-	*-*	-	[17]
	PI 340905-S		quantitative	-	*-*	-	[17]
	PI 155372		quantitative	-	*-*	-	[17]
	Fla7600	breeding line	*Rx3* and *Xv3*		*-*	-	[18]
	Fla 8233	breeding line	quantitative				[35]
	Fla 8517	breeding line	quantitative				[35]
	Fla 8326	breeding line	quantitative				[35]
T4 (1998)	LA 716 (HR)	*S. penneli*	*RXopJ4*	6	*XopJ4*	J350 & J352	[8,36,37]
	PI114490	*S. lycopersicum var. cerasiformae*	quantitative	2,3,10,11	-	C2_Atlg10050 for QTL on chr 11	[21,38]
	PI 128216	*S. pimpenellifolium*	quantitative	-	-	-	[39]
	PI 126932	*S. pimpenellifolium*	quantitative	-	-	-	[39]
	Fla 8233	breeding line	quantitative	11	-	-	[35,38]
	Fla 8517	breeding line	quantitative	3, 11	-	-	[35,38]
	Fla 8326	breeding line	quantitative	11	-	-	[35,38]
X. *gardneri*	LA2533 (HR)	*S. pimpenellifolium*	-	-	-	-	[19]
	LA1936 (HR)	*S. pimpenellifolium*	-	-	-	-	[19]
	PI 128216 (HR)	*S. pimpenellifolium*	-	-	-	-	[19]

**^a^** Tomato species in which corresponding resistant genotype belongs to. **^b^** Resistant genes identified in a respective genotype against specific race. **^c^** Chromosomes in which bacterial spot resistance loci are located in tomato. **^d^** Bacterial effectors present in corresponding *Xanthomonas* race. **^e^** Molecular markers developed for the selection of respective resistance loci in tomato.
